# Applying phylogenetic methods for species delimitation to distinguish B-cell clonal families

**DOI:** 10.3389/fimmu.2024.1505032

**Published:** 2024-12-02

**Authors:** Katalin Voss, Katrina M. Kaur, Rituparna Banerjee, Felix Breden, Matt Pennell

**Affiliations:** ^1^ Department of Quantitative and Computational Biology, University of Southern California, Los Angeles, CA, United States; ^2^ Department of Zoology, University of British Columbia, Vancouver, BC, Canada; ^3^ Bioinformatics Graduate Program, Faculty of Science, University of British Columbia, Vancouver, BC, Canada; ^4^ Department of Biological Sciences, Simon Fraser University, Burnaby, BC, Canada; ^5^ Department of Biological Sciences, University of Southern California, Los Angeles, CA, United States

**Keywords:** B-cell receptor repertoire, B-cell clonal family delimitation, species delimitation, AIRR-seq, somatic hypermutation, benchmarking

## Abstract

The adaptive immune system generates a diverse array of B-cell receptors through the processes of V(D)J recombination and somatic hypermutation. B-cell receptors that bind to an antigen will undergo clonal expansion, creating a Darwinian evolutionary dynamic within individuals. A key step in studying these dynamics is to identify sequences derived from the same ancestral V(D)J recombination event (i.e. a clonal family). There are a number of widely used methods for accomplishing this task but a major limitation of all of them is that they rely, at least in part, on the ability to map sequences to a germline reference set. This requirement is particularly problematic in non-model systems where we often know little about the germline allelic diversity in the study population. Recognizing that delimiting B-cell clonal families is analogous to delimiting species from single locus data, we propose a novel strategy of reconstructing the phylogenetic tree of all B-cell sequences in a sample and using a popular species delimitation method, multi-rate Poisson Tree Processes (mPTP), to delimit clonal families. Using extensive simulations, we show that not only does this phylogenetically explicit approach perform well for the purpose of delimiting clonal families when no reference allele set is available, it performs similarly to state-of-the-art techniques developed specifically for B-cell data even when we have a complete reference allele set. Additionally, our analysis of an empirical dataset shows that mPTP performs similarly to leading methods in the field. These findings demonstrate the utility of using off-the-shelf phylogenetic techniques for analyzing B-cell clonal dynamics in non-model systems, and suggests that phylogenetic inference techniques may be potentially combined with mapping based approaches for even more robust inferences, even in model systems.

## Introduction

**Figure 1:**
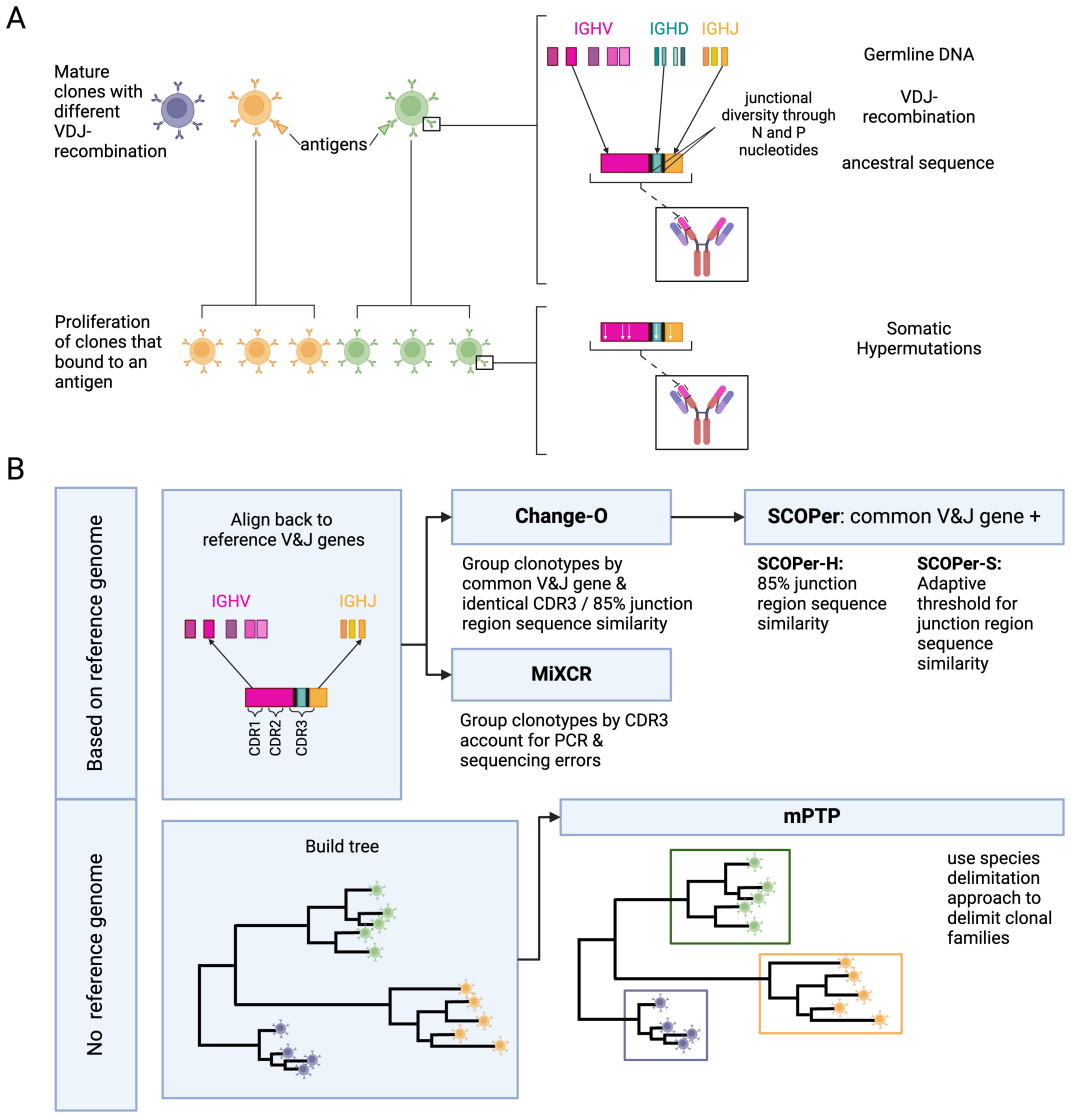
**(A)** Development of B-cell receptors and their sequences. Mature B-cell clones stem from B-cell precursors. Their BCR sequences arise through V(D)J-recombination on the light and heavy chain. During V(D)J-recombination N and P nucleotides are added and removed between the junctions leading to more junctional diversity. Once a mature B-cell binds to an antigen it proliferates into more descendant clones within a clonal family. During proliferation the rearranged IG genes undergo somatic hypermutations. **(B)** Overview of the methods and their requirements. We used two types of methods: methods dependent on a reference genome and methods independent of a reference genome. For the independent method, mPTP (16), we first build a phylogenetic tree of all sequences with RAxML-NG (42). mPTP then fits two Poisson Processes, one for speciation and one for coalescence, to the data and groups the clones accordingly. The reference-genome-dependent methods first align the sequences to a reference genome to find the used V and J genes. This alignment is either done by the methods themselves (MiXCR (26)) or done through IMGT (39). MiXCR delimits the clones by the CDR3 region, accounting for PCR and sequencing errors. Change-O (27) delimits the clones by a common V and J gene and either an identical CDR3 region or a 85% junction region similarity. SCOPer (28) uses the common V and J groups provided by Change-O. The hierarchical method then further groups by 85% junction region similarity. The spectral method calculates an adaptive threshold for junction region similarity. Created in BioRender. Voss, K. (2024) BioRender.com/b54j335.

B-cells and their diverse repertoires of receptors are a central component of the adaptive immune response. Naive B-cells, which have not previously encountered foreign antigens, can become activated upon binding of their B-cell receptors (BCRs) to antigens presented by pathogens (1). Upon activation, these B-cells undergo proliferation and differentiation, ultimately leading to the secretion of antibodies specifically designed to recognize and bind the encountered pathogens (1). These antibodies play a crucial role in the immune defense by either directly neutralizing pathogens or triggering downstream immune responses that lead to pathogen clearance (1). A diverse repertoire of BCRs is necessary to recognize a broad spectrum of pathogens. This diversity is achieved through two primary mechanisms (**Figure 1A**): V(D)J-recombination and somatic hypermutations (SHM). B-cell receptors are composed of two identical heavy chains and two identical light chains. For this study, we concentrate on the heavy chain. The heavy chain locus encompasses V, D, and J genes, and through V(D)J-recombination, one V gene, one D gene, and one J gene are joined together. In the human heavy chain locus we know of approximately 129 V genes, 27 D genes and 9 J genes (2, 3). Consequently the V(D)J-recombination contributes significantly to the vast diversity observed in B-cells. Another big contributor to the diversity of the BCRs is the addition or removal of P and N nucleotides at the junctions of the genes during V(D)J-recombination (1). The parts of the BCRs that bind to antigens are called complementarity-determining regions (CDRs). There are 3 CDRS: CDR1 and CDR2 are encoded in the V-gene, the CDR3 region encompasses part of the V-gene, the junction regions, the D gene and part of the J gene, and is a strong determinant of the specificity of each receptor. Following antigen binding, a B-cell undergoes affinity maturation, a process characterized by clonal expansion and SHM. The point mutations enhance antibody diversity and can lead to the production of antibodies with increased affinity for the antigen (1). A clonal family refers to the collective group of B cells originating from a single V(D)J rearrangement event.

The human body harbors approximately 10^11^ B-cells (4), suggesting a vast array of clonal families. Recent advances in high-throughput sequencing technology have revolutionized the field of BCR repertoire sequencing (5), enabling the analysis of the clonal relationships of BCRs. One of the central challenges in B-cell analysis lies in accurately delineating these clonal families within sequencing data from each individual. As the sequences within a clonal family originate from the same ancestral B-cell, they should not be treated independently in statistical analysis. Only once the clonal families have been identified, is it possible to infer which ones have expanded in response to antigen binding. Subsequent analyses can include examination of V(D)J gene usage, calculation of SHM statistics, quantification of selection during affinity maturation, and inference of the original receptor sequence and identification of the original antigen target [for reviews see (6, 7)]. Furthermore, by tracing the development and diversification of B-cell lineages, it is feasible to identify the specific genetic and structural alterations that give rise to antibodies capable of neutralizing a wide spectrum of pathogenic strains. This identification of broadly neutralizing antibodies serves as the foundation of effective vaccine design against challenging pathogens such as HIV (8, 9).

Rather than approach this problem from scratch, we start by recognizing that the problem of B-cell clonal delimitation bears a close resemblance to a well-studied problem in evolutionary biology: species delimitation. Finding ways to objectively split individuals into species has been a major preoccupation of phylogeneticists for decades and there are a multitude of methods for doing so (10–12). The majority of modern species delimitation approaches use multi-locus approaches, leveraging information from variation in the gene trees between different genomic regions (12). However, in the cases of bacteria and other organisms which do not have (frequent) recombination between loci, researchers often rely on information from the branching structure of a single gene tree to delimit species (13). [Single gene tree approaches have also been used for taxa with limited genomic information (14)]. Broadly speaking, the idea is that the pattern of tree branching *within* a species will be distinct from that of the branching *between* species. In principle, if one has an accurate phylogenetic tree connecting all samples, one could identify the places on the phylogeny where the branching structure transitioned from the between species branching distribution to the within. The most commonly used method that follows this logic is the Poisson-Tree-Process (PTP) approach (15), which as the name suggests uses a Poisson process to model evolutionary branching, both within- and between species. This method has been expanded (16) to allow for background rate variation across the tree in the rate of between-species branching, which we know to be pervasive (17, 18). PTP and *multi-rate Poisson Tree Processes (mPTP)* methods have collectively been used hundreds of times to delimit species in cases such as free-living amoebae (19), Plecoptera (20), radicine pond snails (21), and freshwater mussels (22). And in general, the PTP methods have been found to be superior to closely related alternatives, such as the General-Mixed-Yule-Coalescent method (23), which tends to oversplit taxa (12).

Here we explore whether we can use this method off-the-shelf to delimit B-cell clonal families. The analogy here is if we were to build a complete tree of all B-cell sequences, we should see the footprint of two types of processes — the historical diversification of V genes (24) (analogous to macroevolutionary speciation) and then, following V(D)J recombination, SHM within a clonal lineage (analogous to splitting within a population). Furthermore, there is likely variation among B-cell families in the number of SHM events [even, if just stochastically; see (25)], thus justifying the use of the more general mPTP approach compared to the PTP approach. If mPTP performs competitively to existing approaches, it would present a valuable alternative for analyzing sequences from organisms lacking a reliable reference genome. Additionally, it could be integrated with existing methods to enhance the accuracy of widely used approaches for B-cell clonal family assignment.

In this investigation we will compare mPTP to four state-of-the-art methods that all rely on a reference genome: MiXCR (26), Change-O (27), SCOPer hierarchical (SCOPer-H) (28), and SCOPer spectral (SCOPer-S) (29) (**Table 1**). MiXCR (26) involves an initial alignment of sequences to a reference genome, followed by the assembly of clonotypes based on identical sequences for user-defined gene features like the CDR3 region since it encompasses the majority of the diversity of BCR sequences. By allowing fuzzy matches MiXCR tolerates PCR and sequencing errors (26). Change-O (27) requires a preceding alignment performed by IMGT/HighV-QUEST (30), IgBLAST (31) or iHMMune-align (32). Subsequently, Change-O utilizes these alignments to reconstruct germline sequences and proceeds to group the sequences by the same V gene, J gene, and junction region. The junction region is defined as the CDR3 region plus flanking amino acid residues (29). Typically it is used with a user-defined cutoff delineating the minimum similarity threshold for two junction regions to be considered clonally related. It uses the assumption that sequences sharing highly similar junction regions likely originate from the same clonal ancestor, since it is unlikely that different recombinations result in the same junction region. We elected to use two approaches for Change-O: First we used Change-O with no specified threshold, which results in clusterings where only sequences with the same V-J group and identical junction region are grouped together. And secondly we used it with the threshold of 0.15 which is typically used in studies on human B-cell repertoires (33–36). SCOPer (28) is part of the Immcantation framework specifically designed for the assignment of B-cell clones. In our study, we employed two models of SCOPer: the hierarchical model (SCOPer-H) and the spectral model (SCOPer-S). Both of these models utilize the outcomes generated by Change-O as their input. SCOPer-H is a different implementation of Change-O with the specified cutoff. Therefore in all our results Change-O (0.15) and SCOPer-H are shown together. In contrast to SCOPer-H, SCOPer-S takes an adaptive approach by calculating the optimal cutoff for each group with the same V and J genes and identical junction region length (29). A comparison of the tools’ requirements can be found in **Table 1**. A recent study systematically compared the performance of several tools on both empirical and simulated data (37). The study also evaluated an alignment-free method that does not depend on a reference genome, but this method underperformed relative to others. Change-O with a dissimilarity threshold was identified as the top-performing method in this analysis.

**Table 1:** Comparison of the different tools.

	MiXCR	Change-O (identical)	Change-O (0.15)/SCOPer-H	SCOPer-S	mPTP
aligns sequence to reference genome	✓	✓	✓	✓	
creates groups based on common V and J gene		✓	✓	✓	
compares CDR3/junction region between sequences	✓	✓	✓	✓	
uses a threshold for sequence similarity	✓		✓	✓	
requires a tree					✓

All presented methods have distinct preprocessing procedures and employ varied methodologies to address the clonal family assignment problem. Here we list the specific requirements of each tool and the different approaches to this problem. ✓= yes

We raise the unanswered question: How well does a phylogenetic-based method perform for B-cell clonal delimitation compared to current state-of-the-art methods? By conducting simulations of B-cell repertoires focused on the heavy chain, considering variables such as clonal family count, SHM, and average lineage count per clonal family, we aim to comprehensively measure and compare the performance of the state-of-the-art tools in B-cell analysis to a phylogenetic method. An overview of the methods and a visualization of our pipeline is shown in **Figure 1B**. We adopted a multifaceted approach to assess the performance of each method, employing measures such as the Mean Squared Error (MSE) of the median family size, the number of discerned clonal families, recall/sensitivity, precision, specificity and the F1-score. The MSE and the number of identified families offer insights into overall trends, while recall, specificity and the F1-score, provide a detailed understanding of method performance. The F1-score serves as our primary performance metric. Additionally, we investigated the impact of the tools’ performance on downstream analysis, particularly focusing on ancestral sequence reconstruction. Finally, we compared the phylogenetic-based method to a state-of-the-art method on an empirical IgG repertoire dataset of cattle. This serves as a standardized foundation for future studies delving into B-cell data analysis, providing valuable insights into optimal tool selection under various conditions.

## Materials and methods

### Simulations

To conduct a thorough analysis of the diverse tools under distinct conditions, we systematically simulated B-cell repertoires, manipulating parameters such as SHM and lineage count per clonal family. These simulations were executed with partis (38), a Hidden Markov Model-based framework specifically designed for B- and T-cell receptor sequence annotation. The utilization of partis in these simulations ensures a reliable and standardized platform for assessing the performance of the tools across a spectrum of conditions within the B-cell repertoire. In our study, we used the simulate-from-scratch option within partis to generate a comprehensive dataset comprising 1200 simulated B-cell repertoires. These repertoires were systematically simulated across 24 distinct parameter configurations. Specifically, we simulated 6 SHM rates: 0.001, 0.005, 0.01, 0.05, 0.1, 0.2 (mutation rate per position), encompassing a broad spectrum of mutation scenarios. The true SHM rate is estimated to be 1 in 10^3^ base pairs per cell division (1). This is challenging to rescale for a simulation setup, primarily because the number of cell divisions per sample in nature is variable and not always known. However, by simulating a spectrum of SHM rates, we aim to capture trends in performance across all methods. Antibody sequences may typically exhibit divergence of on average 5-10% from their original germline sequence (1, 6). Hence, we calculated the extent of divergence between our simulated sequences and their true ancestral counterparts to assess the variability. Our examination indicated that the higher SHM rates adhere to this criterion (**Supplementary Figure S1**). Thus, our simulations reflect a realistic degree of somatic hypermutations. This approach allows us to explore how different SHM rates impact the performance of each method and to identify the most suitable method for various scenarios. Similarly, we selected four values for the mean number of leaves per clonal family (10, 20, 50, 100), drawn from a geometric distribution, to explore a range of simulation sizes while ensuring that the total number of sequences remained manageable and did not excessively impact runtimes. For our main simulation setup we chose to simulate 16 different clonal families. We later also explored this parameter by choosing 10, 20 and 50 clonal families. While empirical datasets would typically involve larger scales, our approach allows us to focus on the comparative performance of the tools, with minimal impact on the statistical outcomes, aside from differences in runtime. After observing that all methods tend to oversplit families and result in many singletons, simulated clonal families consisting of one sequence, we decided to remove singletons for our analysis. Since the goal of our study is to test the method’s ability to discern families and not singletons this simplifies the analysis.

In order to further pinpoint the differences between the tested tools we conducted additional simulations without partis, but using an algorithm developed in our lab, across the same parameters. This approach enabled us to simplify the simulations, focusing specifically on scenarios where SHM exclusively impacts the junction regions, excluding the V, D, and J genes within a clonal family. This refinement was particularly motivated by the analytical emphasis of SCOPer on the junction regions. To simulate realistic sequences, we utilized the *ImMunoGeneTics (IMGT)* (39) reference directory, which contains most of the known human V,D, and J genes. For each naive sequence we randomly sampled from the reference genes and joined them together, adding 6 N and P nucleotides. This number was chosen for simplicity and since it does not introduce frameshifts. For the generation of SHM, targeting the junction regions within a clonal family only, we employed the phangorn package (40) in R, leveraging its simSeq function. This function facilitates the simulation of sequences based on a specified phylogenetic tree. This targeted simulation approach allowed us to craft scenarios precisely aligned with the questions and considerations specific to SCOPer’s analytical focus on junction regions.

We additionally created a separate simulation set to test how the methods perform when the sequences do not align well with the reference genomes. To achieve this, we took the original V gene sequences from IMGT (39) and introduced three deletions and three insertions of sizes varying from 1 to 4. We also included point mutations as a parameter, ranging from 20 to 40. Due to the relatively short length of the D and J genes, we did not modify them. The rest of the simulation procedure remained the same as in the previous setup, with 6 N and P nucleotides added at each junction.

### Tools

In this study we evaluated the performance of a phylogenetic method compared to multiple state-of-the-art tools for clonal assignment in B-cells. In the following we explain the approaches and specify the parameters used for each tool in this study.

#### mPTP

mPTP is a single-locus species delimitation method which uses maximum-likelihood and Markov chain Monte Carlo sampling (16). It takes a binary phylogenetic tree T as input. We used Clustal Omega (41) to create the multiple sequence alignment necessary for the tree building. We employed RAxML-NG (42) in our study to infer the phylogenetic tree from the sequence data, aligning with the recommended methodology for implementing the mPTP approach by the authors. When applying mPTP to an empirical dataset, we utilized VeryFastTree (43) for tree building, as RAxML-NG was unable to handle the large number of sequences in the empirical dataset. VeryFastTree provided an efficient alternative, allowing us to process the data while maintaining computational feasibility for such a large-scale analysis. We note that there is a potential trade-off between computational efficiency and statistical accuracy: we expect RAxML-NG (and alternatives, such as IQTree (44)) to obtain a better estimate of the true pattern of historical branching compared to VeryFastTree. However, given that we have a huge number of short sequences, it is very difficult for any method to obtain the correct phylogeny (45) — and it is doubtful that a single solution even exists (46). Furthermore, we are primarily interested in inferring the “backbone” of the phylogeny and not the relationships among sequences within a clonal family. At such, we argue that for our purposes the gain in efficiency is worth the loss in accuracy (see (45) for similar lines of reasoning); this may not be true of other types of problems where inferring the granular structure of the phylogeny is critical [e.g (25)].

The objective of mPTP is to find a binary subtree G of T such that the likelihood of the branch lengths of G fitting an exponential distribution and the branch lengths of each maximal subtree of T formed by the remaining branches fitting an exponential distribution is maximized. Here, G represents the speciation process, while all other maximal subtrees of T represent the coalescent processes. mPTP uses a dynamic programming approach that traverses all nodes of T in postorder traversal. The delimitation with the smallest Akaike Information Criterion score is selected as the final result. To evaluate the confidence of the chosen delimitation, mPTP utilizes an MCMC approach. We used flags –ml and –single as is recommended by the authors.

#### MiXCR

MiXCR is a tool for immune data analysis (26, 47) for diverse downstream analyses, one of them being clone identification. It discerns the clonal families by sequence identity on specific gene features. In our study, we specifically opted to assemble clonotypes based on the CDR3 region, aligning with common practices in real-world analyses. For aligning we used the –preset rnaseq-bcr-full-length flag.

#### Change-O

Change-O is a toolkit with diverse applications in immunogenetics. It depends on an alignment to a reference genome for clonal family assignment. In our study, we chose to align the sequences using IMGT/V-QUEST (48). To streamline this process, we adapted the vquest API provided by the ShawHahnLab (49) for the required output type excel. We used the DefineClones.py script twice: once without specifying a threshold, which resulted in sequences being grouped by their V and J gene and by an identical junction region; and a second time with the commonly used threshold of 0.15, which groups sequences by their V and J gene, and by 85% junction region similarity. This allowed us to compare the performance of strict (identical) and relaxed (0.15 threshold) clonal definitions with Change-O.

#### SCOPer

SCOPer leverages the output of Change-O. There are two models of SCOPer, that we chose to employ: SCOPer-H and SCOPer-S. SCOPer-H is an alternative implementation of Change-O and users must define a threshold. SCOPer-S autonomously determines optimal threshold values for each subgroup identified by Change-O. For our evaluation, we adhered to the default cutoff of 0.15 for SCOPer-H, as suggested by Nouri et al. (28). This threshold is commonly utilized in previous studies on human B-cell repertoires (33–36). Since SCOPer-H and Change-O (0.15) are different implementations of the same algorithm, the results are shown together. For the SCOPer-S model we used the parameter “novj” for the method. It has been shown in previous comparisons that the difference in results between the methods “novj” and “vj” is not very big (37), which we observed on our datasets as well (data not shown).

### Metrics for assessing performance

To comprehensively evaluate the performance of all tools, we used various measures. In all our analyses, we opted to exclude singletons, which are derived clonal families containing only a single sequence, in order to reduce noise and because they are disregarded in real-life analyses as well. For a broad overview and indirect assessment, we computed the MSE of the Median Family Size. This metric serves to determine whether the identified families align closely in size with the actual families, providing valuable insights into the overall accuracy of family size assignments across the evaluated methods. To find the cause of large MSEs we also counted the number of families that the methods derived for each simulation and compared it to the real number of clonal families. This helped us to understand whether the methods were over or under splitting the clonal families.

For more direct and more interpretable metrics, we computed precision, recall, and the F1-score. In our evaluation, analogous to other studies with similar assessments (29, 50–52), we defined True Positives (TP), True Negatives (TN), False Positives (FP), and False Negatives (FN) according to the following criteria:

For each sequence *x_i_*:

TP: # of sequences from the same family as *x_i_* that are correctly identified as being in the same family

TN: # of sequences from a different family as *x_i_* that are correctly identified as being in a different family

FP: # of sequences from a different family as *x_i_* that are incorrectly identified as being in the same family

FN: # of sequences from the same family as *x_i_* that are incorrectly identified as being in a different family

We then calculated the precision, recall, specificity and F1-score (harmonic mean of precision and recall) for each *x_i_*. Recall/Sensitivity answers the question: Of all sequences that were clustered together, how many actually belong to the same family? Precision answers the question: Of all the sequences belonging to the same family, how many were correctly clustered together? Specificity calculates how many of all sequences belonging to different clonal families were clustered together. The F1-score is the harmonic mean of precision and recall and therefore increases when reducing the instances where a single clonal family is divided into multiple groups and the cases where multiple clonal families are combined into a single group. For all quantities we averaged them over all sequences to end up with one value per simulation.


$$\text{Recall}/\text{Sensitivity }x_{i}:\frac{TP_{x_{i}}}{TP_{x_{i}}+FN_{x_{i}}}$$

$$\text{Precision }x_{i}:\frac{TP_{x_{i}}}{TP_{x_{i}}+FP_{x_{i}}}$$

$$\text{Specificity }x_{i}:\frac{TN_{x_{i}}}{TN_{x_{i}}+FP_{x_{i}}}$$

F1‐score xi:2TPxi2TPxi+FPxi+FNxi

#### Ancestral sequence

Our downstream analysis consists of the evaluation of the ancestral sequence reconstruction. For each inferred clonal family comprising more than two sequences we first aligned the sequences and subsequently constructed a phylogenetic tree using RAxML-NG. Ancestral sequences were then reconstructed using RAxML-NG with the GTR model. As input we used both an unrooted tree as returned by RAxML-NG and a tree rooted using the midpoint root, which roots the tree halfway between the longest two tips. We then calculated the Hamming distance between the inferred sequence and the correct naive sequence from the simulations. As a control, we repeated this process for correct families, recognizing that a correct family does not necessarily lead to the correct ancestral sequence, owing to uncertainty in the ancestral reconstruction itself.

### Empirical dataset

To evaluate mPTP on an empirical dataset, we applied it to a cattle immunoglobulin repertoire from a study by Safonova et al. examining vaccine responses (53). The data used for the empirical data analysis is available at https://github.com/yana-safonova/great_cattle_ab_repertoire and has been preprocessed by Safonova et al. For our analysis, we selected individual 14007 and analyzed its repertoire at timepoints day 0 and day 21. In the original study clonal families were delimited by V and J genes and an identical CDR3 region. We compared the number of singletons inferred by both their delimitation and by mPTP, as well as the overall clonal family size distribution. Finally, we analyzed whether each sequence was grouped into a similarly sized clonal family by both approaches.

## Results

### Change-O (0.15)/SCOPer-H outperform all other methods

**Figure 2:**
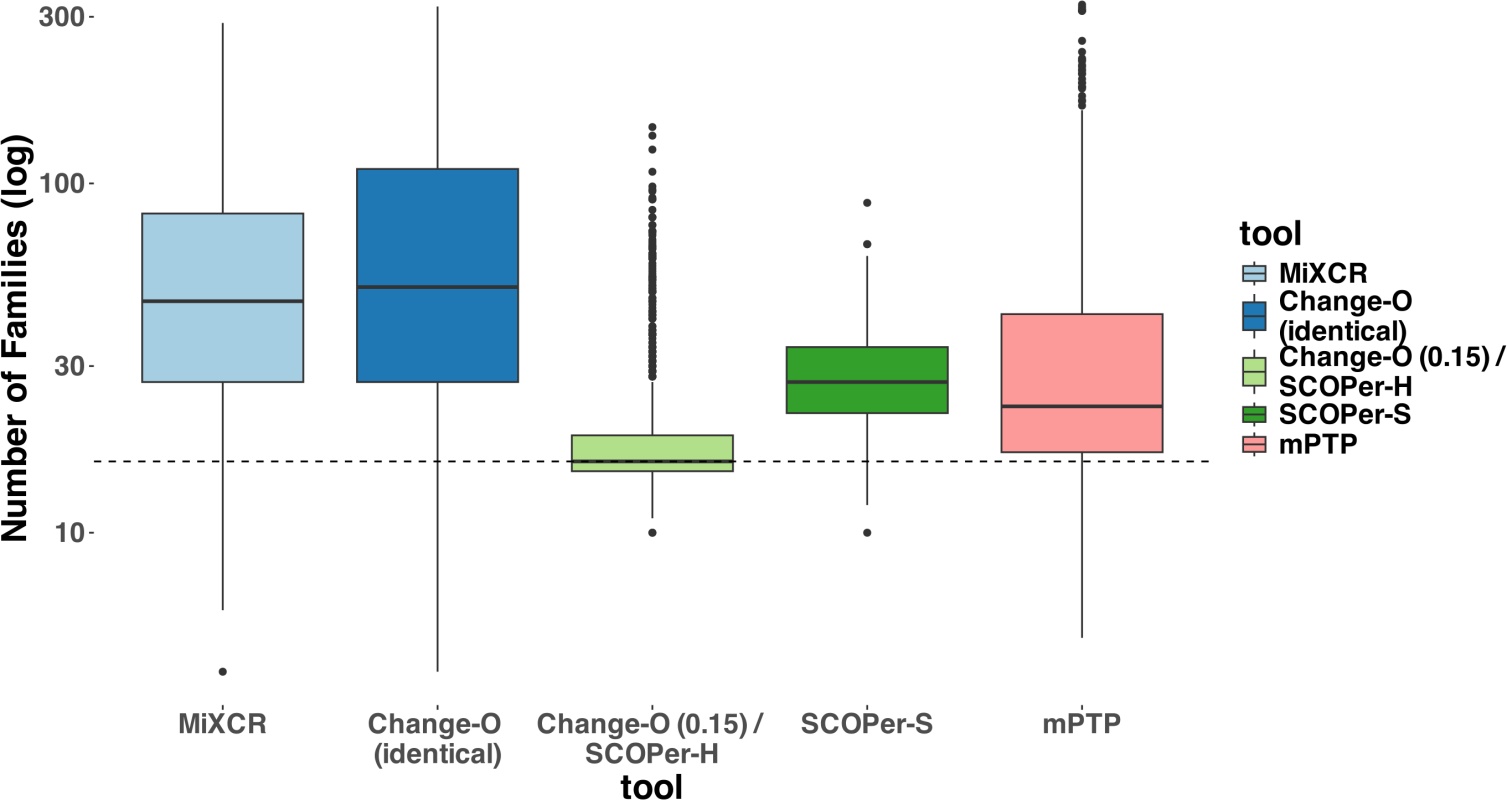
Number of Clonal Families (log scale) discerned by the different tools. For this analysis we removed discerned singletons. We counted the number of clonal families that the methods discerned for each simulation and compared it to the correct number of clonal families (in this case 16). The dashed line represents the correct number of clonal families (ground truth). We can clearly see that all methods consistently return a higher number of families, indicating the subdivision of one correct family into multiple. Change-O (0.15)/SCOPer-H on average perform the best.

In our evaluation we considered multiple measures across the parameters and tools. For all tools except mPTP, an increase in the SHM rate resulted in fewer sequences being analyzed (**Supplementary Figure S2**). We classified the missing samples as singletons— clonal families with only one sequence. To mitigate the distortion caused by singletons, which are often disregarded in real-life analysis (36), we excluded all singletons from our analysis. Initially, we assessed the MSE of the median family size for all tools. On average, Change-O (0.15)/SCOPer-H exhibited the best performance based on the MSE metric (**Supplementary Figure S3**). To further interpret this finding, we analyzed the number of clonal families identified by each method compared with the actual number of families. Across all methods we observed a consistent pattern of overestimating the numbers of clonal families, even after removing the discerned singletons as seen in **Figure 2**. This indicates a tendency for all methods to oversplit clonal families. Change-O (0.15)/SCOPer-H gets closest to the correct number of clonal families across all leaf and SHM configurations. SCOPer-S has the second best performance on this measure in most leaf configurations, but is outperformed by mPTP for the three lowest SHM values. It is notable that on average SCOPer-S has the smallest interquartile range whereas mPTP has the largest. MiXCR and Change-O (identical) perform similarly to each other and poorly relative to the other tools.

**Figure 3:**
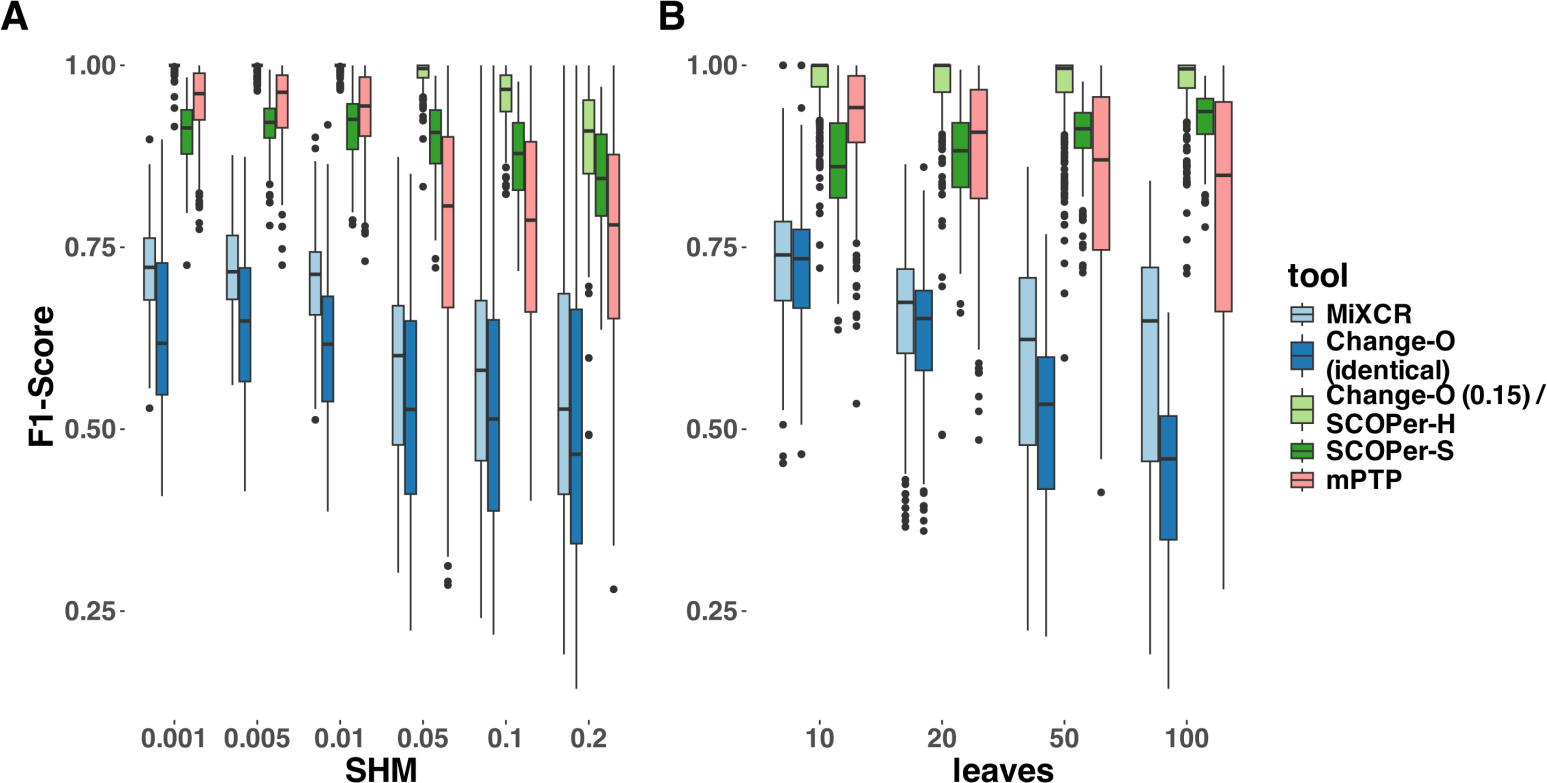
F1-Score yielded by the different methods. For this analysis we removed singletons. **(A)** different SHM rates **(B)** different average number of leaves per clonal family. The F1-score is the harmonic mean of precision and recall, a score of 1 meaning perfect precision and recall.

To provide a more nuanced assessment of the tools’ performances, we calculated the F1-score for each sequence—representing the harmonic mean of precision and recall—and then averaged these scores across all sequences in each simulation. Across all parameter configurations, Change-O (0.15)/SCOPer-H consistently outperformed all other methods by a substantial margin (**Figure 3**, **Supplementary Figure S4**). mPTP and SCOPer-S emerged as contenders for the second-best performance. SCOPer-S exhibited superior performance at higher leaf and SHM configurations, whereas mPTP demonstrated better performance at lower leaf and SHM configurations (**Supplementary Figure S4**). MiXCR and Change-O (identical) again demonstrated the poorest performances, with MiXCR slightly outperforming Change-O (identical). The same pattern emerges when examining recall/sensitivity (**Supplementary Figure S5**). While most tools maintain a specificity of 1 across the majority of simulations, mPTP occasionally groups multiple clonal families together, leading to false positives and slightly reducing its specificity in those instances (**Supplementary Figure S5**). We have similar explanations for the poor performance of both MiXCR and Change-O (identical): MiXCR groups sequences solely based on identical matches and then allows for fuzzy matches to accommodate PCR and sequencing errors. However, the SHM rate is high and likely surpasses what MiXCR’s fuzzy matching can accommodate. As a result, MiXCR tends to oversplit clonal families. Similarly, Change-O (identical) initially groups sequences by VJ-genes and then by identical junction regions, failing to group sequences from the same clonal family with SHM in the junction region. Consequently, Change-O (identical) also tends to oversplit clonal families. The same patterns were consistently observed across simulations with varying numbers of clonal families (10, 20, and 50) (**Supplementary Figure S6**). Given that the primary simulation with 16 clonal families captures the trends seen in all other simulations, it serves as an appropriate representation of the broader dynamics for our analysis.

Our analysis clearly identified hierarchical clustering with a cutoff at 0.15 to be the best method in our selection of tools across parameters. We explored various thresholds for sequence similarity which revealed a consistent trend: higher thresholds led to improved performance for SCOPer-H (**Supplementary Figure S7**). Across the range of thresholds tested, all options yielded superior or comparable results compared to SCOPer-S. This contradicted the anticipated superiority of SCOPer-S over SCOPer-H. SCOPer-S was designed to enhance accuracy by dynamically calculating an optimal threshold for similarity within each VJ-group obtained from Change-O, rather than employing a fixed threshold for all groups. However, our findings deviate from the anticipated outcomes and contradict the results of the tool’s authors (28). To delve deeper into this discrepancy, we designed a simulation set specifically targeting the junction region, as this is the focal point of SCOPer’s analysis for both models. We incorporated the parameter “junction region length” into our setup, considering the developers’ indication that the performance of the hierarchical model depends on the junction region length, which we had not analyzed in our previous simulations. For these simulations we randomly sampled V,D and J genes from the IMGT (39) reference directory and joined them together, adding 6 N and P nucleotides in between to not cause a frameshift. We also only simulated SHM at the junction region, to analyze the effect it has on the performance of SCOPer. Analysis of the F1-score revealed a notable decline in performance for Change-O (0.15)/SCOPer-H at an SHM rate of 0.2. Further exploration pinpointed this decline to junction region lengths of 70 and above (**Supplementary Figure S8**). We were not able to replicate the finding that the performance of SCOPer-H declines for shorter junction regions (28), and in most of our new simulations SCOPer-H still outperformed SCOPer-S (**Supplementary Figure S8**).

To validate our findings and ensure they were not biased by our simulations, we also ran both models on subsets of the simulation data provided by Nouri et al. (29). The results confirmed that SCOPer-H generally outperforms the spectral model in typical scenarios (**Supplementary Figure S9**). In Nouri et al.’s comparison of the spectral and hierarchical model their validation primarily relied on a limited simulation setup and empirical data (28). In their validation process using empirical data, the emphasis was on confirming highly homogeneous discerned clonal families. This favors SCOPer-S because of its tendency to oversplit clonal families, resulting in each discerned family being highly homogeneous. Although they later conducted an extensive simulation, they only evaluated the performance of SCOPer-S. Our simulations revealed that SCOPer-S is overly stringent, resulting in the oversplitting of clonal families (**Figures 2**, **3**; **Supplementary Figure S9**). Interestingly, in their related simulation study Balashova et al. observed the opposite issue, finding that SCOPer-S tends to group multiple clonal families together, making it the worst-performing method in their study (37). This discrepancy could stem from the limited number of clonal families simulated in our study. Given the small number of families, it is unlikely that two clonal families share the same VJ-junction configuration, which could cause SCOPer-S to calculate a higher threshold and oversplit families. Regardless, SCOPer-S seems to perform poorly in both contexts, suggesting that it may not be a reliable option for real-world data analysis.

### mPTP as a new alternative not reliant on a reference genome

Across a majority of parameters we demonstrated that mPTP outperforms all other methods but Change-O (0.15)/SCOPer-H. Particularly noteworthy is its superior performance compared with SCOPer-S, which shares a similar approach to mPTP but relies on additional information from a reference genome. For low SHM rates, mPTP has the second best performance on average across all tools. This is particularly striking because it is the only method that does not require any information except the sequences. To explore the SHM rates in a more biologically meaningful context, we calculated the distance of the sequences to their ancestral sequences. In nature, it is estimated that the average divergence between antibody sequences and their original germline sequence is around 5-10% (6). Our simulations cover a variety of sequence divergences, including ones with an average of 5-10% (**Supplementary Figure S1**). Our main goal was to examine the performance of the tools across the parameter space to infer trends. mPTP performs better than other methods across all scenarios. As mPTP appears to be a promising alternative in our simulation setting, we aimed to evaluate its performance in situations where methods dependent on a reference genome fail. To do so, we assessed the performance of the tools on a dataset where a reliable reference genome is not available. We achieved this by creating a simulation set with “fake” V genes, by introducing insertions, deletions, and mutations to the known V gene sequences from IMGT (39). We then applied all methods to this simulation set using the same parameters as before. We observed that all the tools relying on a reference genome did not return a substantial number of input sequences in their results (**Supplementary Figure S10**). While we had already noticed a pattern of missing samples with an increase in the SHM rate in the original simulations (**Supplementary Figure S2**), this pattern was magnified with the fake V genes. mPTP, not being dependent on a reference genome, consistently returned all input sequences (**Supplementary Figure S10**). To evaluate the overall performance, we classified the missing samples as singletons and calculated the F1-Score with singletons included. For the three lower SHM rates, mPTP outperforms all other methods (**Supplementary Figure S11**). However, starting at a SHM rate of 0.05, the performance of mPTP decreases significantly. This pattern was also observed in the original simulations (**Figure 3**). By examining the number of singletons per simulation, we found that this decrease in performance is due to over splitting (**Supplementary Figure S12**). At a SHM rate of 0.05 or higher, mPTP struggles to correctly differentiate between sequence differences caused by different V(D)J recombination events and those caused by SHM. Our analysis revealed mPTP to be a valuable alternative to other methods, particularly when the organism of interest lacks a robust reference genome. This makes mPTP a valuable tool for analyzing B-cell repertoire data in diverse contexts, including species with poorly characterized genomes or non-model organisms. Consequently, researchers can leverage mPTP to gain insights into clonal relationships and dynamics without being hindered by limitations associated with reference genome availability or quality.

### Ancestral sequence reconstruction

**Figure 4:**
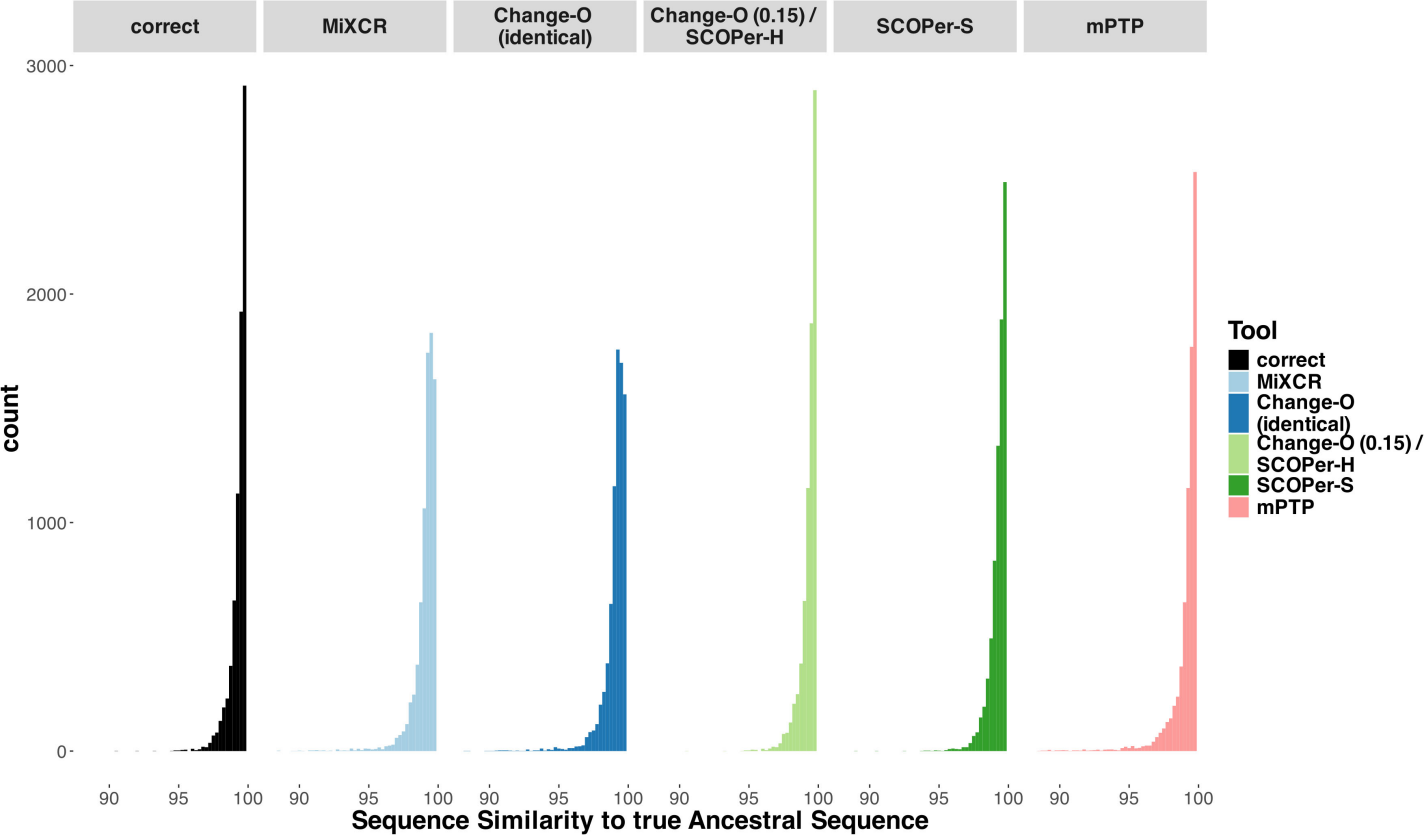
Sequence Similarity between the real ancestral sequence and the derived ancestral sequence based on the clonal families discerned by the methods. If all 5 methods discerned a specific family the only difference being the amount of sequences in a family, we considered it in this analysis. The “correct” column acts as a point of comparison, using the correct sequences of the clonal family for ancestral sequence reconstruction.

The reconstruction of the ancestral sequence from sequences within clonal families is a vital aspect of repertoire analysis. We wanted to evaluate the extent to which errors in clonal family assignment impact ancestral sequence reconstruction, a common downstream inference. For each method, we assessed the ability to reconstruct ancestral sequences from all identified clonal families comprising more than two sequences. This reconstruction process relied on phylogenetic trees constructed from the sequences of each discerned family. We used RAxML-NG (42) for constructing all trees and for reconstructing the ancestral sequence. We explored two different approaches for reconstructing the ancestral sequence: firstly, utilizing the unrooted tree returned by RAxML-NG, and secondly, rooting the tree using midpoint rooting. This second approach positions the root at the midpoint between the two longest branches. Subsequently, we compared the inferred ancestral sequences to the known ancestral sequences and calculated the Hamming distance. As a point of comparison, we repeated this process for the correct families. As expected, our findings align with previous results: the distribution of sequence similarity for Change-O (0.15)/SCOPer-H closely mirrors that of the correct families (**Figure 4**). Following closely are SCOPer-S and mPTP. This underscores the significant impact of method selection on downstream analysis of repertoire sequence data. Our analysis indicates that utilizing the midpoint root yields superior results for ancestral sequence reconstruction across all methods (**Supplementary Figure S13**).

### Application of mPTP on empirical data

We tested mPTP on empirical datasets by applying it to a cattle immunoglobulin repertoire from a study by Safonova et al. that investigated vaccine responses (53). This study analyzed the IgG repertoires of 204 Black Angus calves across four time points during the vaccination process. We selected a random individual (14007) and ran mPTP on their repertoire sequences at two timepoints: day 0 (the day of the first vaccination) and day 21 (three weeks after the first vaccination and the day of the booster vaccination). The dataset is available via the corresponding study’s GitHub repository, and we utilized the preprocessed data where reads were merged and aligned to an existing reference genome. This alignment allowed us to compare their clonal family assignments to those generated by mPTP. In the Safonova et al. study, clonal families were identified by shared V and J gene segments and identical CDR3 regions, which corresponds to the “Change-O (identical)” method used in our simulations. For the sequence alignment, we employed Clustal Omega (41), as in our simulations. Due to the large number of sequences (>300,000, see **Table 2**), RAxML-NG could not be used for phylogenetic tree construction, so we opted for VeryFastTree (43).

**Table 2:** Percentage of inferred singletons at each time point by method.

Time Point	Total Sequences	mPTP singletons	Change-O singletons	shared singletons
Day 0	421,288	39.5%	35.5%	21.7%
Week 3	311,248	35.1%	28.6%	17.3%

**Figure 5:**
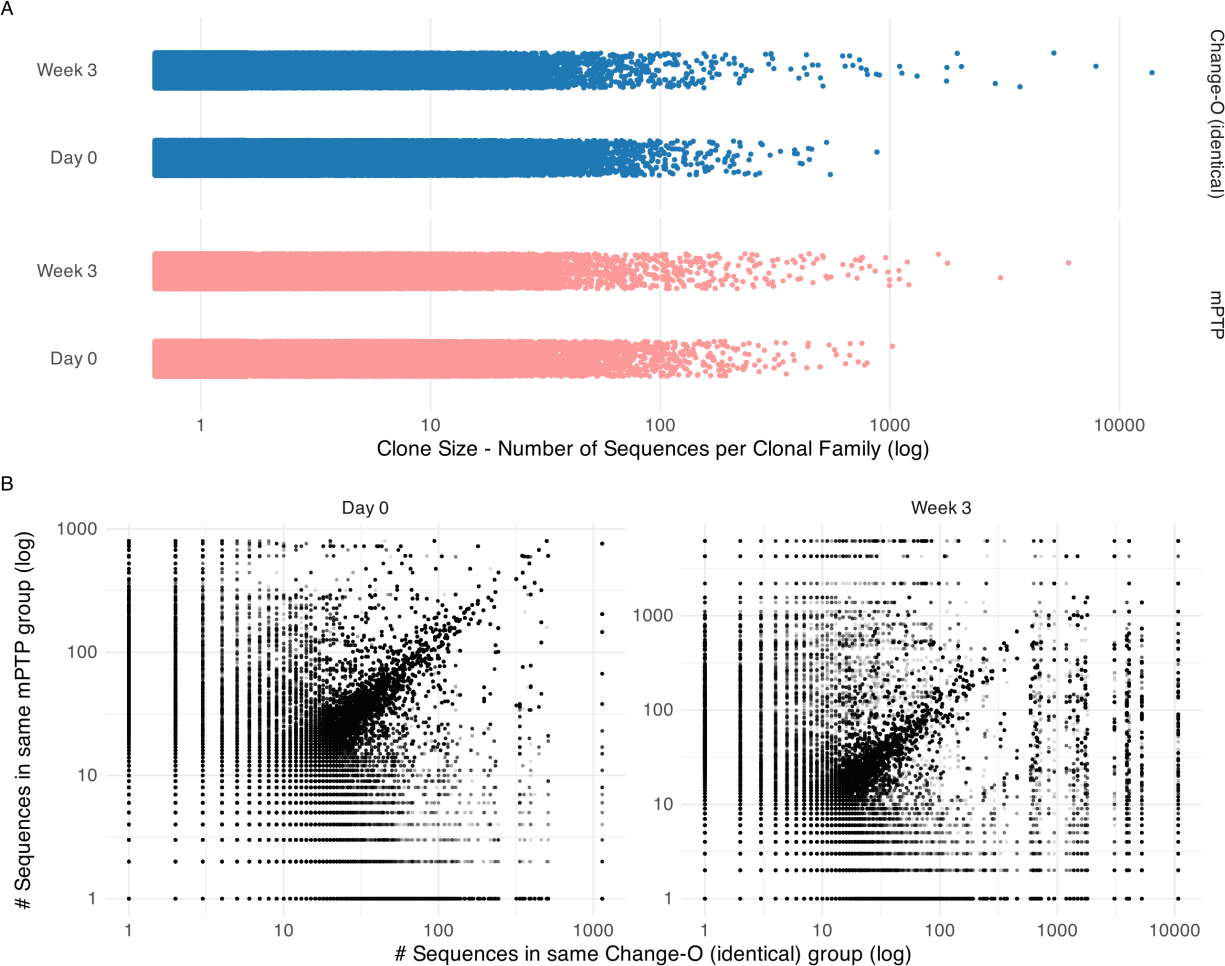
Clonal family size analysis on cattle repertoire. **(A)** Clonal family size distribution across time points day 0 and week 3 for mPTP and Change-O (identical). Both tools capture a trend of bigger clonal families in week 3. **(B)** Comparison of inferred clonal family size for each sequence. Here we calculated the size of the clonal family that the sequence was assigned to by mPTP and by Change-O and plotted them against each other on a log scale. We can observe a diagonal, showing that the tools tend to group sequences into similar size clonal families.

After constructing the tree, we ran mPTP and plotted the inferred clonal family sizes (**Figure 5A**). A trend of larger clonal families was observed after the three-week period, aligning with expectations that certain clones would expand in response to vaccination. This same trend was noted in the clonal families inferred using Change-O (identical) (**Figure 5A**). Both methods returned a substantial number of singletons for both time points, many of them shared (**Table 2**).

We further looked at individual sequences to determine whether they were grouped into clonal families of similar sizes by mPTP and the published clone assignments (**Figure 5B**). While some discordances were present, a clear linear correlation emerged, indicating that mPTP’s clonal family inferences were consistent with the reference-based assignments. This demonstrates that mPTP is applicable to empirical data for clonal family assignment. Our analysis of this AIRR (adaptive immune receptor repertoire) dataset highlights the potential of mPTP for clonal family assignment. Although the repertoire still required preprocessing—such as alignment to an existing reference database for generating informative multiple sequence alignments—mPTP eliminates the need for a highly reliable reference genome in the actual clonal family assignment process. For non-model organisms, where reference genome issues, including naming conventions, can impede accurate clonal inference, mPTP offers significant advantages. Additionally, our analysis showed that both mPTP and Change-O (identical) infer a big number of singletons. While a majority of these singletons are shared between the two methods, there are notable discrepancies where sequences classified as singletons by one method are grouped into larger clonal families by the other. The clear diagonal trend in **Figure 5B** suggests that mPTP could also serve as a validation tool for clonal inferences made by other methods, such as Change-O. Given these findings, we believe that mPTP has a promising future in clonal family inference for both organisms with well-curated reference genomes and those without.

## Discussion

With the widespread adoption of BCR repertoire sequencing, understanding the evolutionary relationships of B-cells has become increasingly feasible. However, accurate delimitation of B-cell clonal families is essential for any meaningful analysis. Numerous tools have been developed to tackle this challenge, employing diverse approaches. Our comparative analysis of four state-of-the-art tools revealed Change-O (0.15)/SCOPer-H as the optimal choice for delimiting B-cell clonal families in organisms with reliable reference genomes, such as humans and mice. Change-O (0.15)/SCOPer-H effectively accounts for both V(D)J-recombination and SHM utilizing a reference genome, making it well-suited for model organisms. Additionally, we found mPTP, a native phylogenetic method for species delimitation, to be effective in delimiting B-cell clonal families across various scenarios. Notably, mPTP does not rely on a reference genome, making it particularly valuable for analyzing non-model organisms lacking a robust reference genome. When applied to an empirical dataset, mPTP identifies clonal families of sizes comparable to those determined by Change-O (identical) and captures similar trends. These findings invite further exploration into the integration of traditional methods with phylogenetic approaches like mPTP to enhance the accuracy of B-cell clonal family inferences. mPTP could serve as a valuable complement to currently employed methods. We also suggest that it would be straightforward, at least in principle, to integrate mPTP into established pipelines and protocols for B-cell repertoire analysis [e.g (26, 27)]; mPTP is available as open-source software under the GNU Affero 3 license.

We recognize that mPTP’s effectiveness as a B-cell clonal family delimitation method depends on the quality of the multiple sequence alignment of all sequences. When sequences are not properly curated the resulting alignments can be less informative, often containing many gaps. However, it is important to note that other clonal delimitation methods are also susceptible to issues caused by poor alignments, particularly when relying on alignment to reference genes. Thus, alignment quality remains a critical factor across all methods. In our investigation, we concentrated on the accuracy of the tools rather than other aspects like computational time. All methods demonstrated similar processing speeds in our simulations. However, computational time might be an important consideration for analyses involving empirical data. Our investigation into the downstream effects of clonal assignment on ancestral sequence reconstruction revealed that the choice of clonal assignment tool significantly influences the accuracy of ancestral sequence inference. This underscores the importance of selecting the most appropriate tool for clonal family assignment, especially in the context of vaccine design and other downstream applications. In conclusion, we found that the phylogenetically explicit method of using mPTP serves as a valuable alternative to current clonal family assignment techniques, especially in non-model organisms where germline reference data may be limited. This approach paves the way for integrating traditional mapping-based methods with phylogenetic techniques like mPTP to achieve more robust clonal family assignment in B-cell research, even within model systems.

## Data Availability

Publicly available datasets were analyzed in this study. This data can be found here: https://github.com/yana-safonova/great_cattle_ab_repertoire.
