# Supplementary material for: Applying phylogenetic methods for species delimitation to distinguish B-cell clonal families
Source: Front Immunol. 2024 Dec 2;15:1505032. doi: 10.3389/fimmu.2024.1505032 (PMC11646844; doi:10.3389/fimmu.2024.1505032)
Supplement: Supplementary file 1 [file DataSheet1.pdf]

Applying phylogenetic methods for species delimitation to distinguish B-cell clonal families

Katalin Voss[1], Katrina M. Kaur[2], Rituparna Banerjee[3], Felix Breden[4] & Matt Pennell[1,5,*]

[1]*Department of Quantitative and Computational Biology, University of Southern California, USA*
[2]*Department of Zoology, University of British Columbia, Canada*
[3]*Bioinformatics Graduate Program, Faculty of Science, University of British Columbia, Canada*
[4]*Department of Biological Sciences, Simon Fraser University, Canada*
[5]*Department of Biological Sciences, University of Southern California, USA*
[*]Corresponding author: mpennell@usc.edu

Supplementary Material

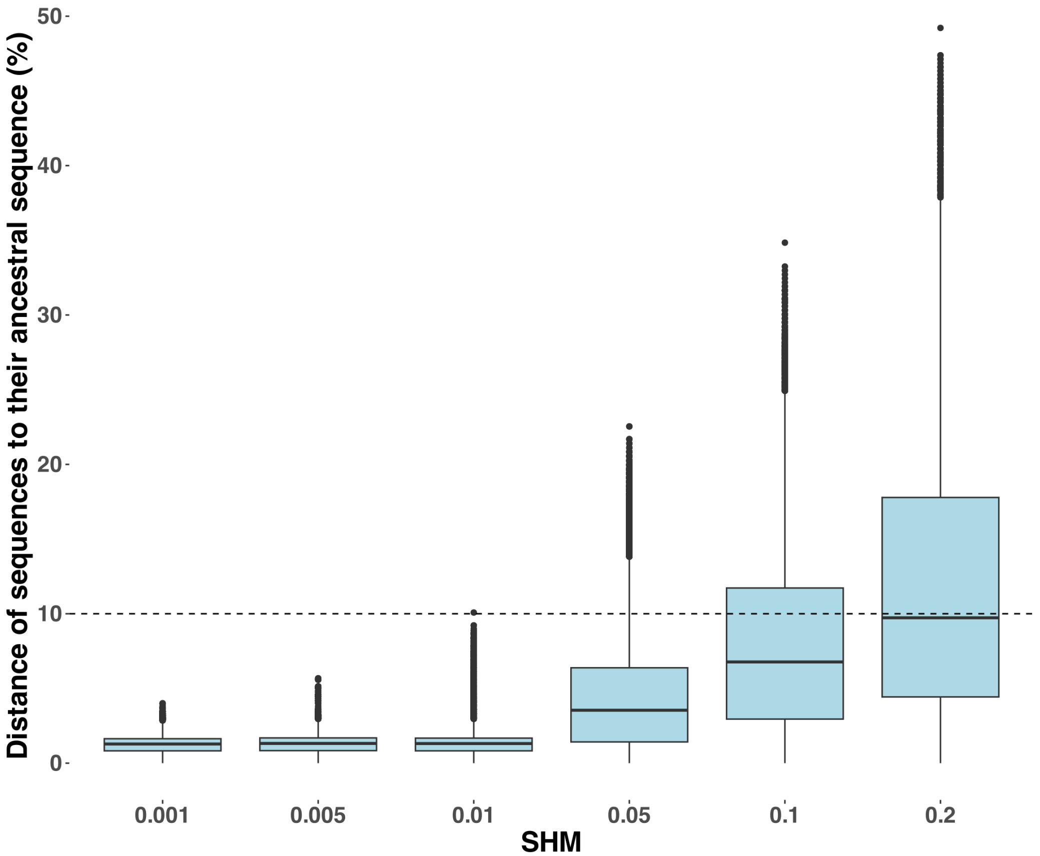

Figure S1: **Sequence Divergence between the simulated sequences and their ancestral sequence**. The dashed line is at 10%, which is a known level of divergence in real sequences.

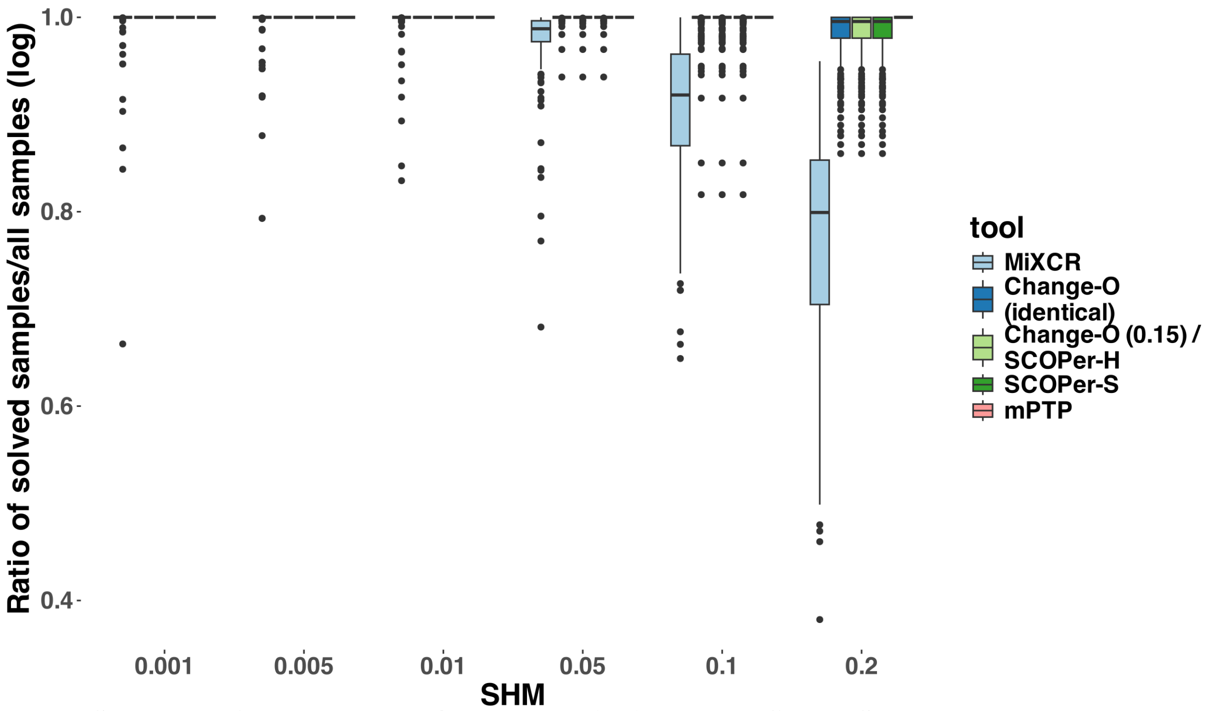

Figure S2: **Ratio of solved samples/all samples (log) across different SHM rates.** For this analysis we counted the amount of sequences that the methods assigned to a clonal family and divided that by the number of all sequences from the input.

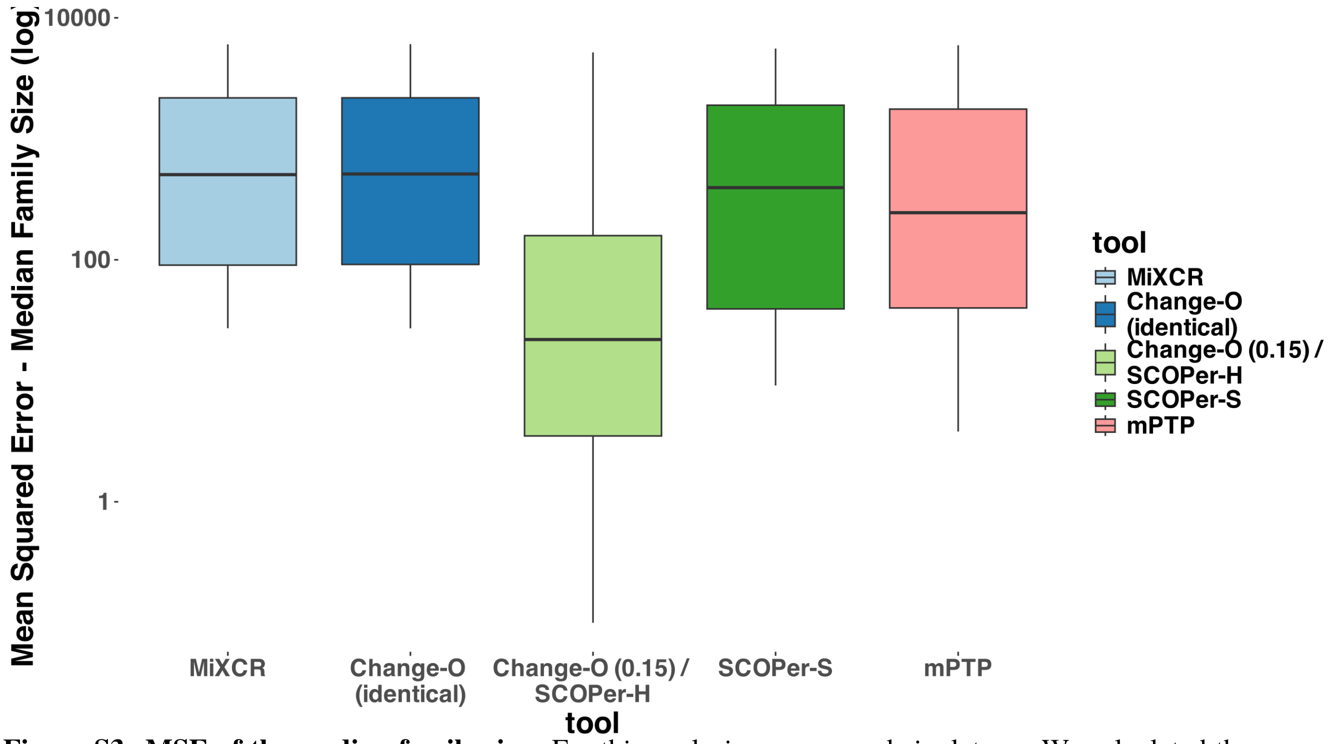

Figure S3: **MSE of the median family size.** For this analysis we removed singletons. We calculated the median family size of the true clonal families and compared them to the median family size of the derived clonal families for each method.

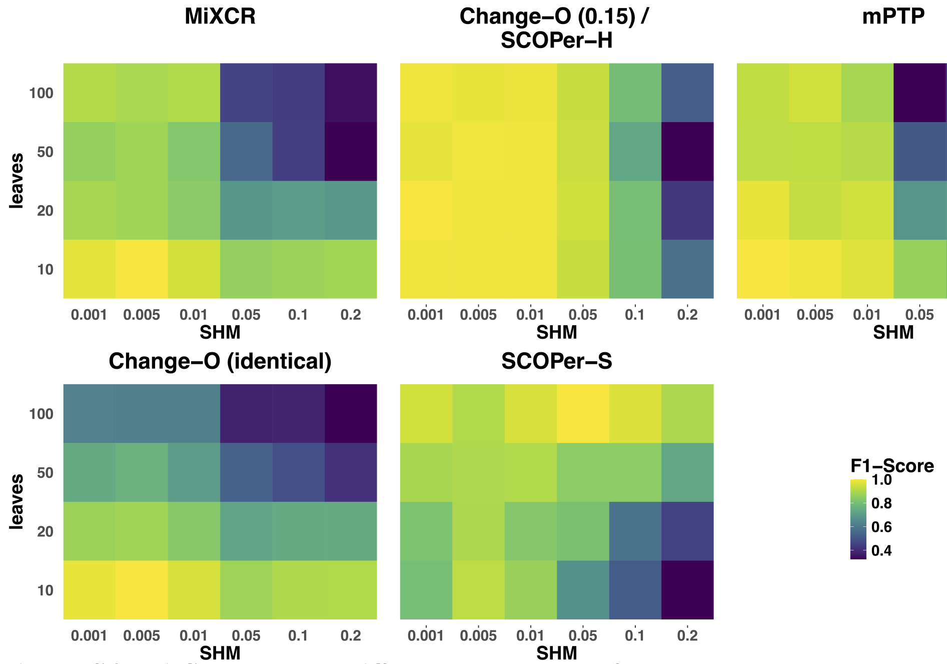

Figure S4: **F1-Score across different parameters for all methods.** For this analysis we removed singletons. The F1-score was calculated by taking the average of all simulations with the specific leaf and SHM configuration.

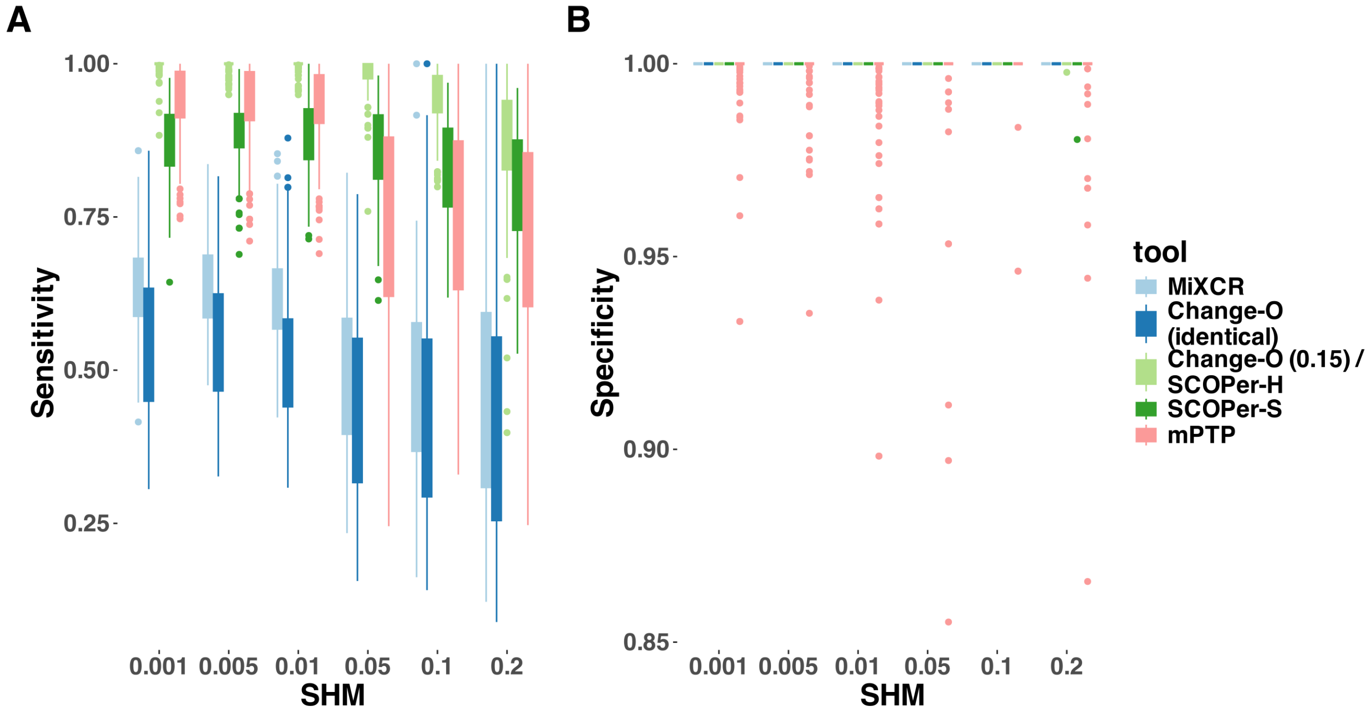

Figure S5: **Sensitivity and Specificity across different parameters for all methods.** For this analysis we removed singletons. Both values were calculated by taking the average of all simulations with the specific leaf and SHM configuration.

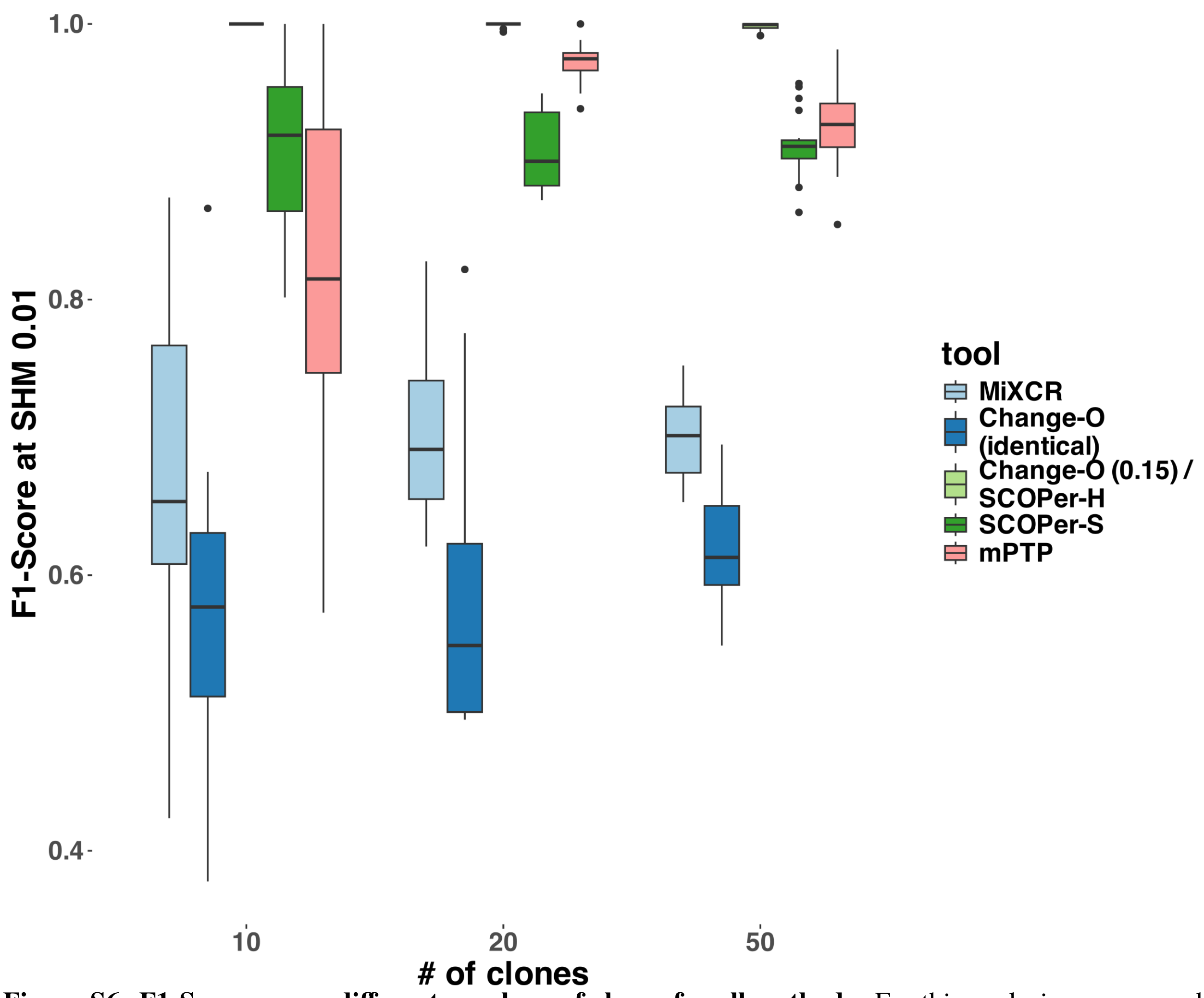

Figure S6: **F1-Score across different numbers of clones for all methods.** For this analysis we removed singletons. The F1-score was calculated by taking the average of all simulations with the same number of clones.

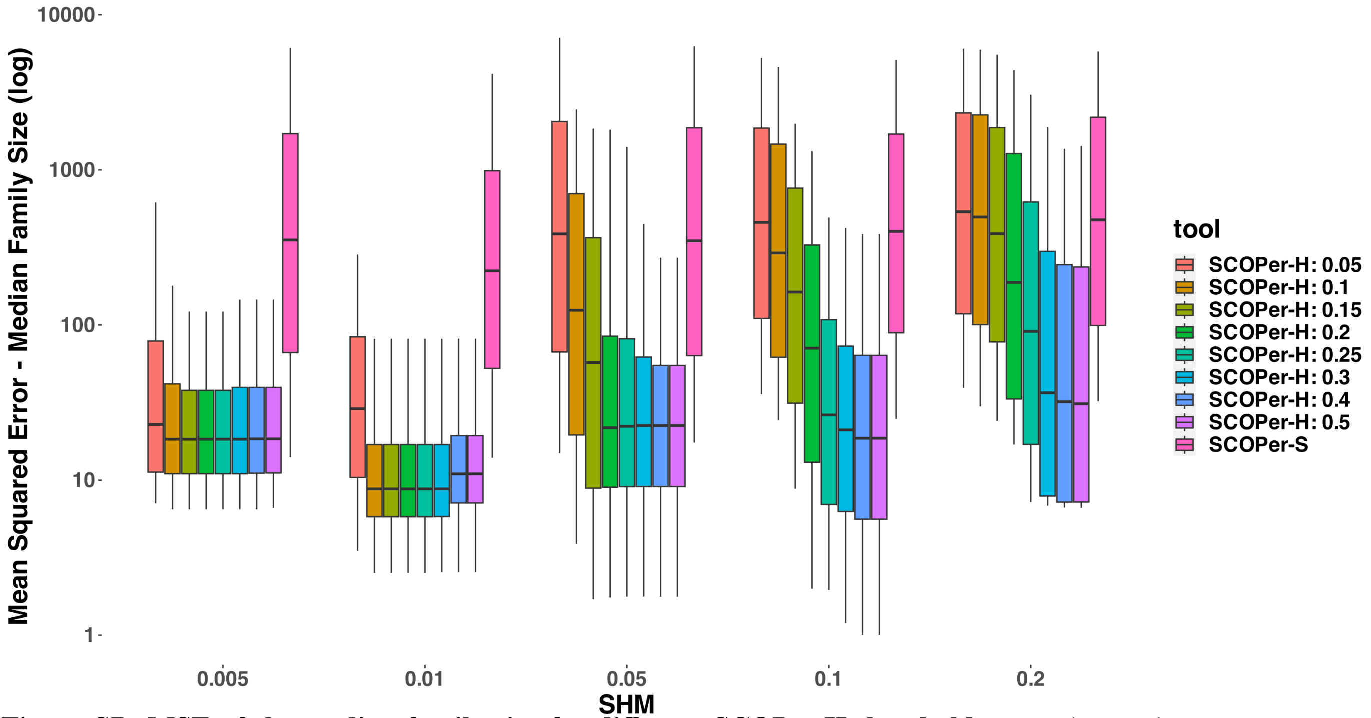

Figure S7: **MSE of the median family size for different SCOPer-H thresholds.** For this analysis we removed singletons. SCOPer-H: 0.15 is the one used in all other analyses.

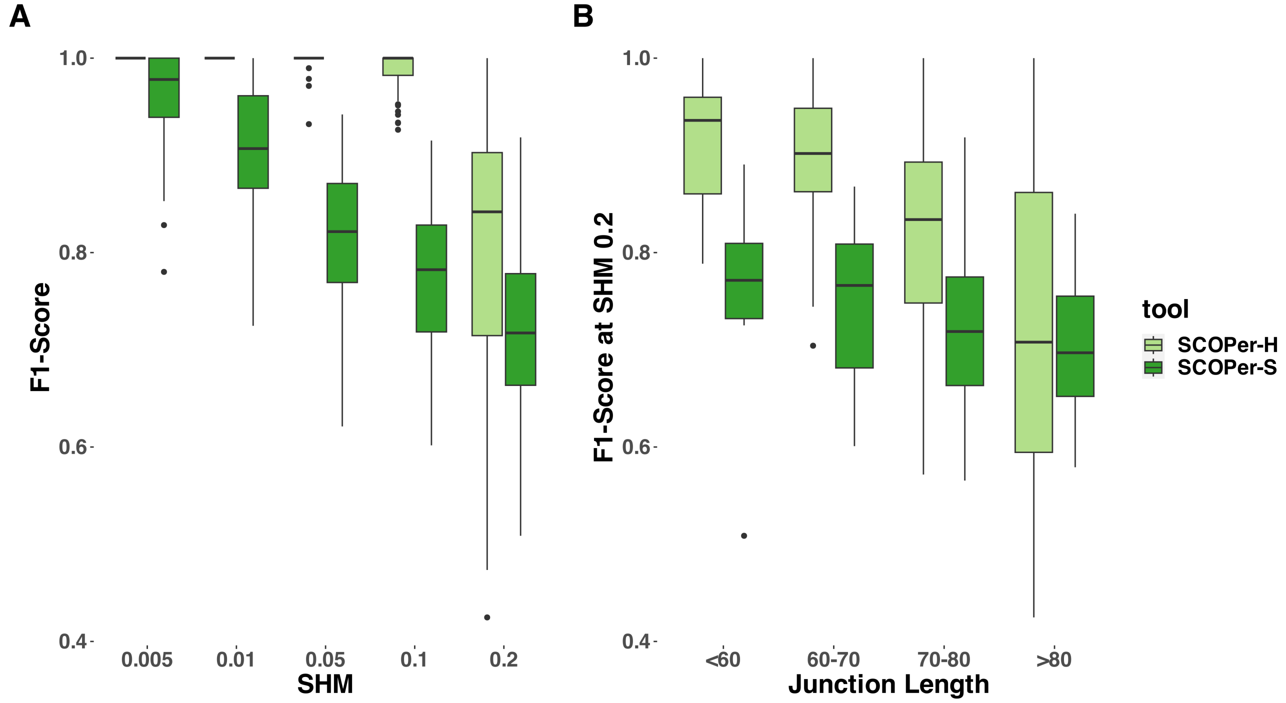

Figure S8: **F1-Score across different parameters for SCOPer-H and SCOPer-S**. For this analysis we removed singletons. A) different SHM rates B) different junction region lengths (nt). The junction region length was calculated by SCOPer.

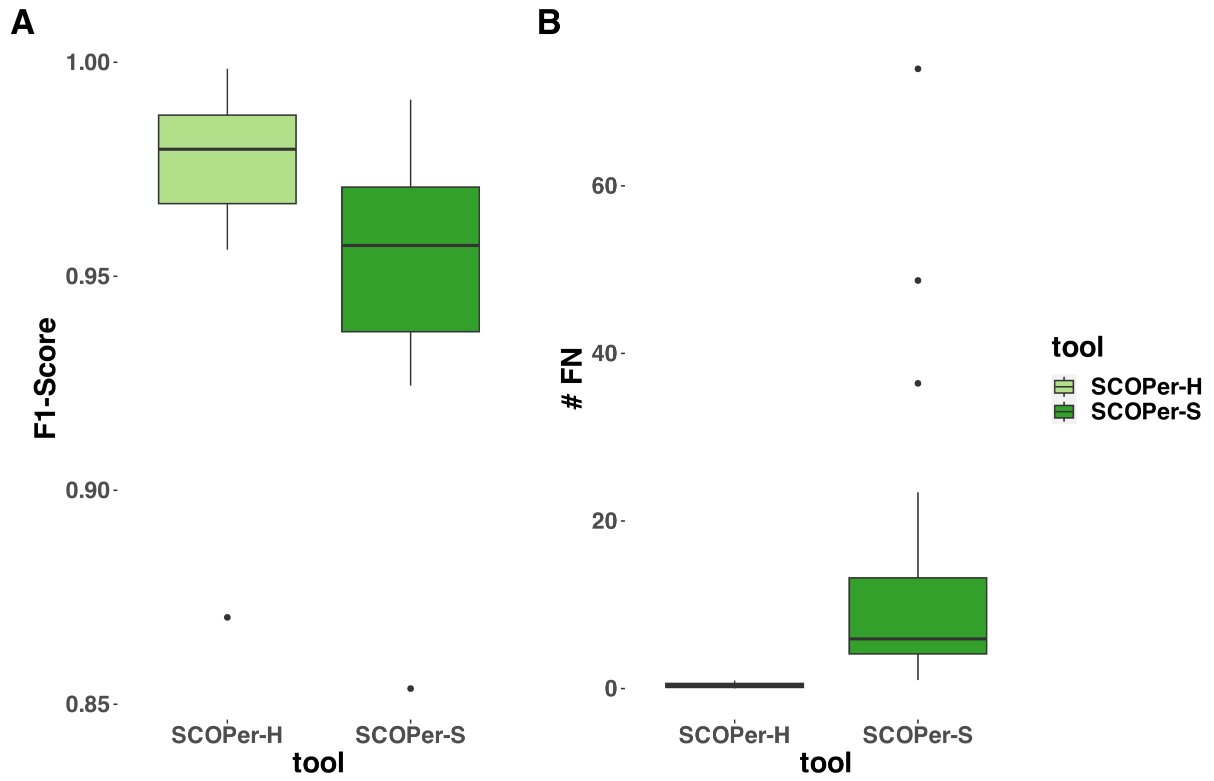

Figure S9: **Evaluation of a subset of simulations provided by Nouri et al. [?]** A) F1-score for SCOPer's hierarchical and spectral model B) number of False Negatives for SCOPer's hierarchical and spectral model. For each simulation we took the 20,000 first results and evaluated them.

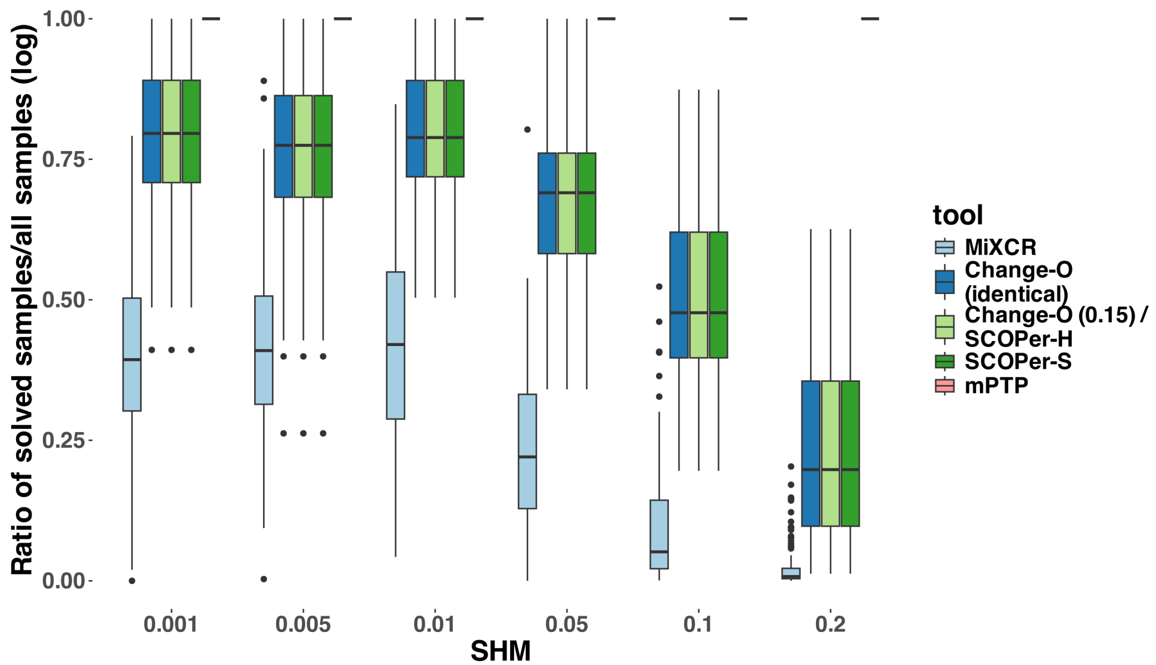

Figure S10: **Ratio of solved samples/all samples (log) across different SHM rates in simulations with "fake" V genes.** For this analysis we counted the amount of sequences that the methods assigned to a clonal family and divided that by the number of all sequences from the input.

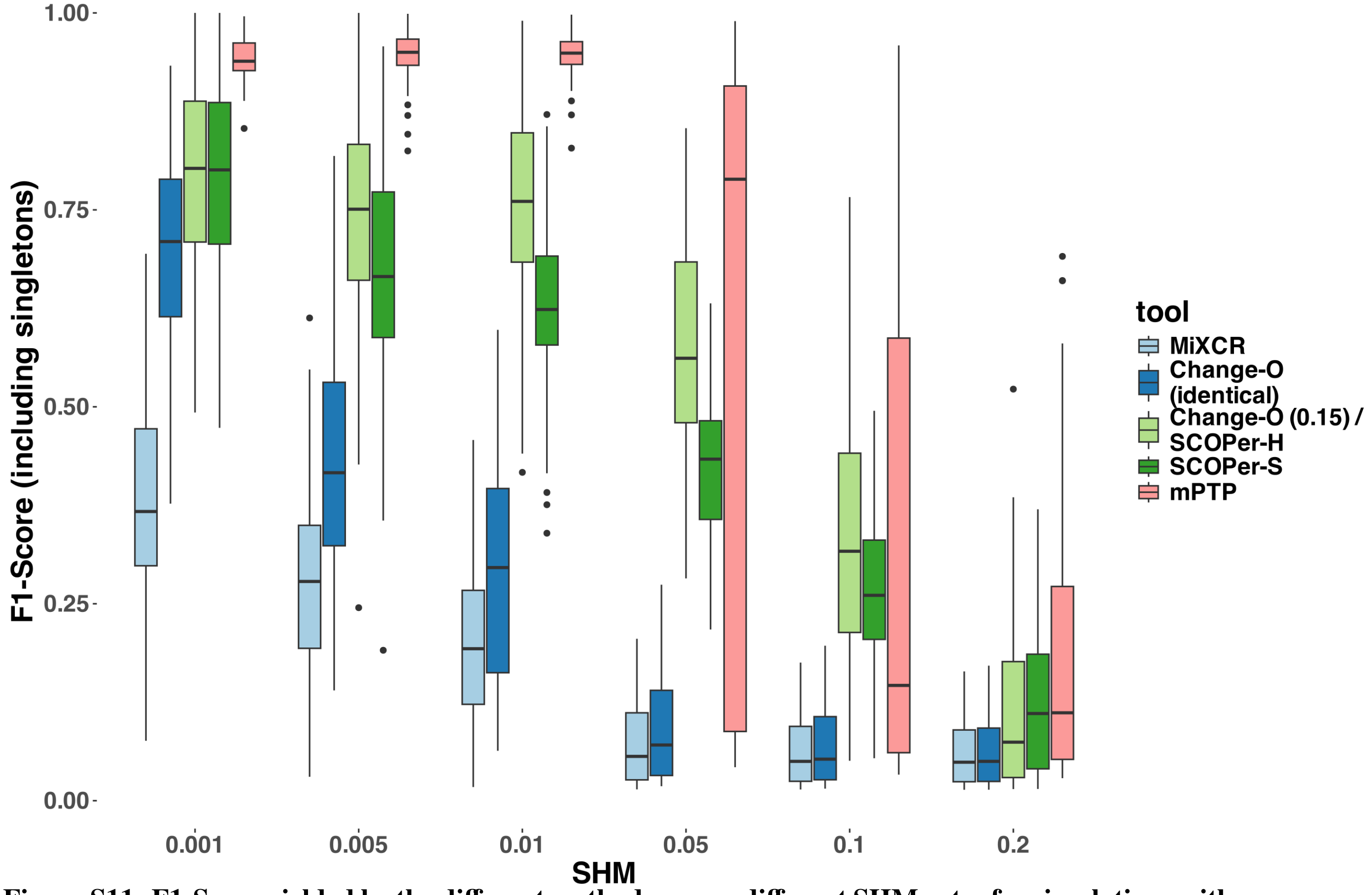

Figure S11: **F1-Score yielded by the different methods across different SHM rates for simulations with "fake" V genes (includes singletons)** The F1-score was calculated by taking the average of all simulations with the specific SHM configurations.

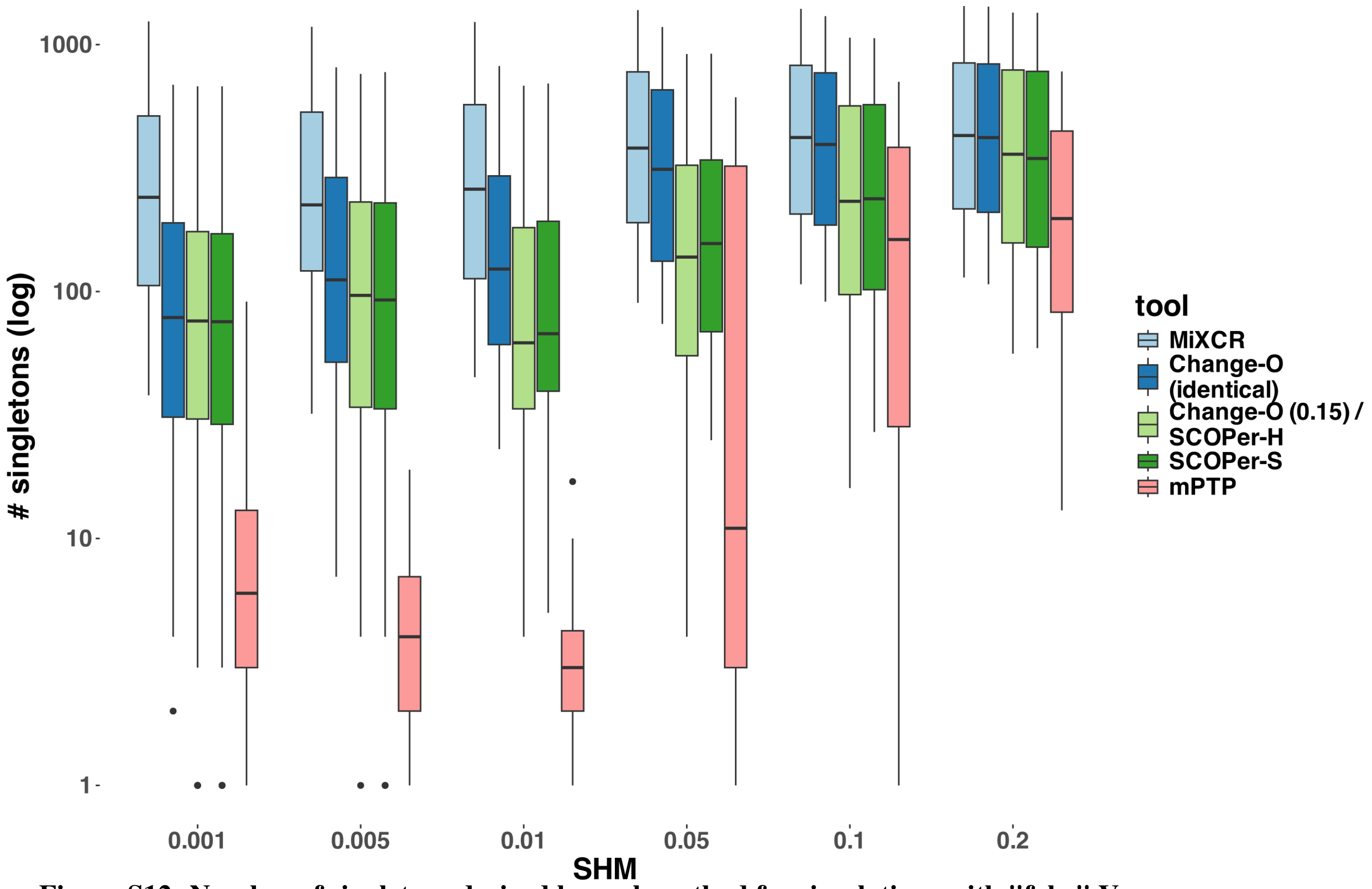

Figure S12: Number of singletons derived by each method for simulations with "fake" V genes

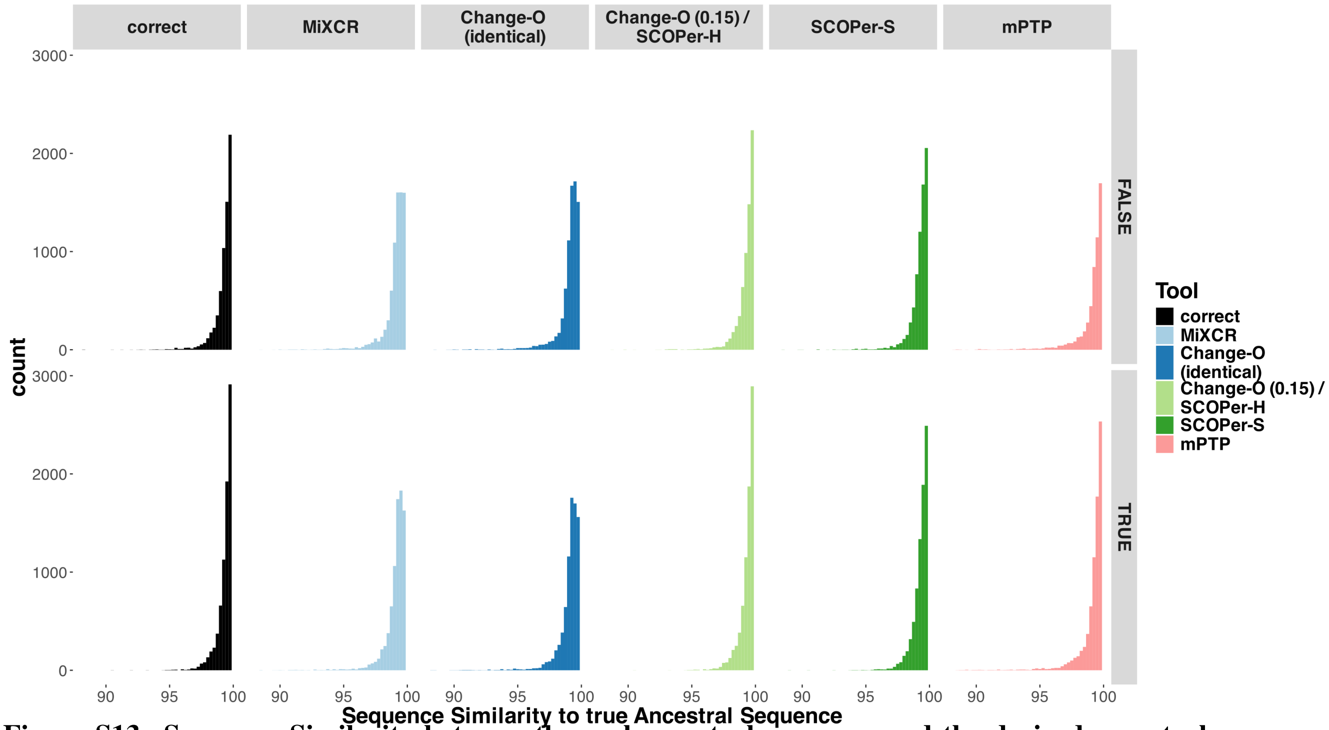

Figure S13: **Sequence Similarity between the real ancestral sequence and the derived ancestral sequence** based on the clonal families discerned by the methods split between using the unrooted tree provided by RAxML-NG (FALSE), and rooting at the midpoint (TRUE)
